# Mechanisms of Strain-Induced Interfacial Strengthening of Wet-Spun Filaments

**DOI:** 10.1021/acsami.1c25227

**Published:** 2022-03-30

**Authors:** Tianyu Guo, Zhangmin Wan, Yan Yu, Hui Chen, Zhifeng Wang, Dagang Li, Junlong Song, Orlando J. Rojas, Yongcan Jin

**Affiliations:** † Jiangsu Co-Innovation Center of Efficient Processing and Utilization of Forest Resources, and Jiangsu Provincial Key Lab of Pulp and Paper Science and Technology, Nanjing Forestry University, Nanjing 210037, P. R. China; ‡ Bioproducts Institute, Department of Chemical and Biological Engineering, Department of Chemistry and Department of Wood Science, The University of British Columbia, 2360 East Mall, Vancouver, British Columbia V6T 1Z3, Canada; § College of Material Science and Engineering, Nanjing Forestry University, Nanjing 210037, P. R. China; ∥ Testing Center, Yangzhou University, 48# Wenhui East Road, Yangzhou 225002, P. R. China; ⊥ Department of Bioproducts and Biosystems, School of Chemical Engineering, Aalto University, P.O. Box 16300, FI-00076 Aalto, Finland

**Keywords:** interfacial strengthening, wet drawing, axial orientation, reversible torsion, carbon nanotube

## Abstract

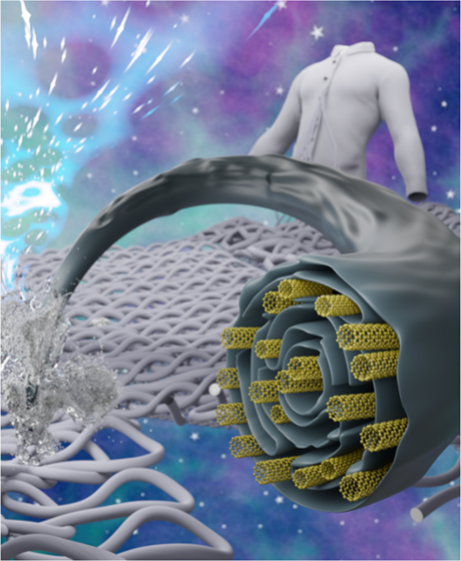

We investigate the mechanism of binding of dopamine-conjugated carboxymethyl cellulose (DA-CMC) with carbon nanotubes (CNTs) and the strain-induced interfacial strengthening that takes place upon wet drawing and stretching filaments produced by wet-spinning. The filaments are known for their tensile strength (as high as 972 MPa and Young modulus of 84 GPa) and electrical conductivity (241 S cm^–1^). The role of axial orientation in the development of interfacial interactions and structural changes, enabling shear load bearing, is studied by molecular dynamics simulation, which further reveals the elasto-plasticity of the system. We propose that the reversible torsion of vicinal molecules and DA-CMC wrapping around CNTs are the main contributions to the interfacial strengthening of the filaments. Such effects play important roles in impacting the properties of filaments, including those related to electrothermal heating and sensing. Our findings contribute to a better understanding of high aspect nanoparticle assembly and alignment to achieve high-performance filaments.

## Introduction

Nature builds responsive and adaptive structures that amplify the properties of materials through the use of component modularity combined with highly ordered structuring.^1^ To note one example, the outer cell wall layer of wood fibers (specifically, the S2 layer in the secondary wall) exhibits remarkable stiffness and strength, which is explained by the hierarchical arrangement of cellulose microfibrils.^2^ This hierarchical structure includes a tilt orientation of cellulose fibrils relative to the longitudinal cell axis (the microfiber angle), which largely determines the stiffness of the system. At a low microfiber angle, the lamellae between each fibril tend to be small, which leads to a tight structure. Upon axial stretching, the microfibers are straightened and resist interfacial shear instead of an early slide between neighboring building blocks. From an atomic-scale perspective, the lack of orientation of pyranoid rings induces steric effects, contributing to sliding resistance and facilitating a process whereby contiguous molecular chains become locked. Overall, the stiffness of plant cells can largely benefit from such structural effects.^3^ Meanwhile, compared with the S2 layer, the middle lamella, with relatively loose microfibrillar packing, requires a larger reorientation under stress, which means early dislocation and sliding tend to happen early under strain. Consequently, the tensile strength in the middle lamella is around 10 MPa, which is far lower than that of the S2 layer.^4−6^

Inspired by the above observations, we expand on the opportunity to improve the mechanical performance of man-made materials, for example, by strengthening the interfacial stability of building blocks present in structured filaments. This can be achieved if one gains a better understanding of the interfacial behavior and dislocation motion of neighboring constituents.^7,8^ In this context, synthetic carbon materials, such as carbon nanotubes (CNTs), are known to be strong and stiff (tensile strength of 13–53 GPa and Young’s modulus of 0.4–5 TPa).^9−11^ Unfortunately, such outstanding properties can hardly be expanded to the macroscale, primarily due to CNT entangling and overlapping, compromising the interfacial ability to resist shear stress.^7,12^ Bai et al. addressed such challenges by bundling ultralong CNTs, leading to a tensile strength of over 80 GPa.^13^ Kim et al. further utilized polydopamine to enhance the interfacial strength of graphene fibers, which effectively improved adhesion between the graphene layers. Using this approach, the elastic modulus of composite fibers reached 110 GPa, nearly 10 times that of neat graphene fibers.^14−16^ Li et al. applied the combination of wet spinning and wet stretching to regulate graphene layers with a highly ordered structure, enhancing the interactions between adjacent graphene sheets. As a result, the mechanical strength of these highly crystallite graphene fibers reached 3.4 GPa.^15^ The described effects use low energy, which is in contrast to other techniques normally used for filament formation, especially those that rely on melting and chemical vapor deposition (CVD).^14,16^ While stretching and alignment of building blocks have been demonstrated for their benefits, their adoption requires well-dispersed precursor suspensions; otherwise, they would impair the effective assembly of building blocks, leading to weakening the strength of interfaces. This is most evident in CNT suspensions that tend to aggregate in water.^17−19^ In this vein, DNA,^20^ proteins,^21^ and polymers^22^ have been shown as effective dispersants of CNTs through noncovalent interactions, for example, by adsorption and wrapping. They lead to a well-dispersed suspension that can be further used in molecular self-assembly.^23^

Previously, we showed that CNT can be conveniently dispersed in an aqueous colloidal suspension using dopamine-conjugated carboxymethyl cellulose (DA-CMC). Moreover, upon wet spinning, tough composite filaments were obtained (76 MJ m^–3^). Therein, dopamine acted as a CNT binder^24^ while carboxymethyl cellulose (CMC) formed the matrix for filament formation.^25^ The DA-CMC filaments displayed a strain-hardening ability under plastic deformation, indicating that the external strain induced axial orientation of the building blocks. To improve strength and stiffness, wet spinning with subsequent stretching was proposed as an efficient, low-energy approach.^26^ Unfortunately, the mechanisms that explain the interfacial strengthening of filaments, such as those relevant to our previously reported DA-CMC/CNT system, have remained open for elucidation. Herein, our goal is to address this knowledge gap, following the guidance of molecular dynamics simulation and confirmatory evidence from experimental data, to lead to an improved performance of structures based on the given building blocks. As such, we study the effects of wet-spinning and drawing on the stiffness of hybrid filaments. The results led to wet-spun filaments displaying a tensile strength and elastic modulus that surpass those measured for all reported cellulose/CNT-based filaments.^27−30^ Meanwhile, acknowledging the differences in composition, we find that the specific strength is higher compared to that obtained by the flow-assisted assembly of nanocellulose.^31^ Finally, we reveal the main interactions and interfacial and structural effects that contribute to the performance of the obtained materials.

## Experimental Section

### Materials

Carboxymethyl cellulose (CMC, sodium form, *M*_w_ = 700 000 g mol^–1^, degree of substitution, DS = 0.9, Aldrich), dopamine (DA, 98%, 189.6 g mol^–1^), *N*-hydroxysuccinimide (NHS), 1-(3-dimethylaminopropyl)-3-ethylcarbodiimide hydrochloride (EDC), and calcium chloride were all purchased from Sigma-Aldrich (Milwaukee). Single-walled carbon nanotubes (CNT, P3-SWNT, diameter: 1–2 nm, length: 1–3 μm) were obtained from Carbon Solutions Inc., CA.

### DA-CMC/CNT Suspensions

DA-grafted CMC (DA-CMC) was prepared by EDC/NHS coupling, as described earlier.^32,33^ In short, 1-ethyl-3-(3-(dimethylamino)propyl)carbodiimide hydrochloride (EDC, 5.0 mmol) was first added to 100 g of 1 wt % CMC solution and magnetically stirred at 60 °C for 1 h. *N*-Hydroxysuccinimide (NHS, 5.0 mmol) and dopamine hydrochloride (DA, 5.0 mmol) were then added and stirred at 22 °C under a nitrogen atmosphere for 24 h. After removing the unreacted agents via dialysis, the resulting DA-grafted CMC solution was lyophilized overnight. DA-CMC was added to a high concentrated CNT solution at a given loading (50 wt %) under 30 min ultrasonication and extra mechanical stirring for 2 h. Following this, the obtained DA-CMC/CNT suspensions were used to prepare the filaments. Neat CNT suspension (without DA-CMC) was also produced as a reference.

### Filament Synthesis (Wet-Spinning and Wet-Drawing)

**Figure 1:**
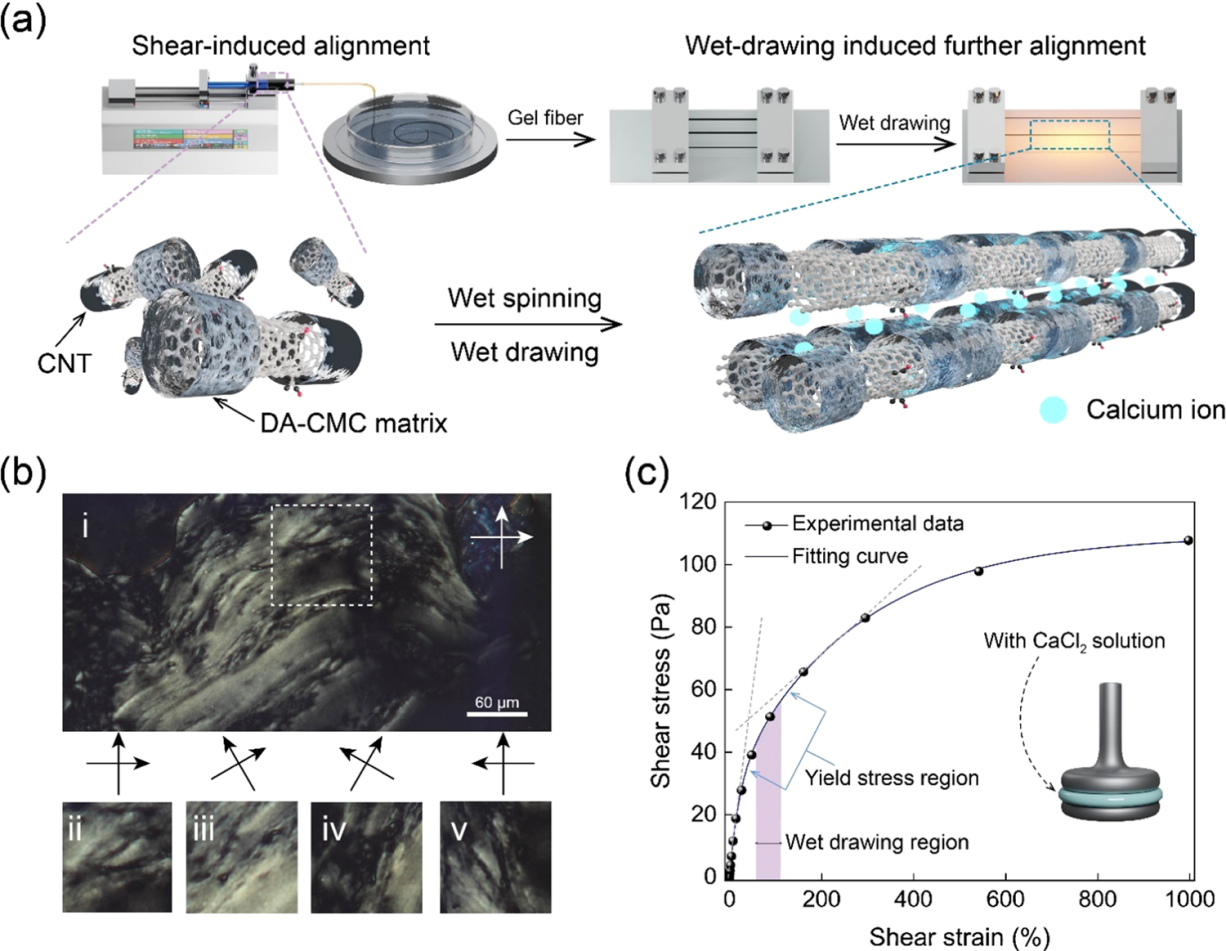
Axial orientation and assembly by controlled wet spinning and drawing: (a) Schematic illustration of the wet-spinning process followed by wet drawing. The carbon nanotubes wrapped with DA-CMC are shown in a disordered orientation in the suspension, but they became aligned after wet spinning and wet drawing. (b) Schlieren texture of the DA-CMC/CNT suspension through polarized optical microscopy (POM) at rotation angles of 0, 30, 60, and 90°. (c) Viscoelasticity (strain sweep) of the DA-CMC/CNT suspension in the presence of CaCl_2_.

The conditions used for wet spinning followed our previous report.^23^ In short, as shown in Figure 1a, the DA-CMC/CNT and neat CNT spinning suspensions were loaded into a 5 mL plastic syringe using a spinning nozzle (PEEK tube with a diameter of 0.5 mm) and continuously injected into a rotating bath driven by a pump operated at 4.2 mL min^–1^. Ethyl alcohol was used as coagulation fluid. The wet-spun DA-CMC/CNT gel filaments were transferred into the calcium chloride (CaCl_2_) solution (5 wt %) and dipped for 2 h. Subsequently, the gel filaments were towed out from the solution for further wet drawing. A continuous strain was achieved by securing the filament in a controlled universal testing machine (SANS, CMT4202, China) that used two clamps located between the stretcher and operated at a constant displacement of 1 mm min^–1^ (see Video S1). Then, 4 cm of the wet thread was loaded and gradually stretched at a speed of 15 mm min^–1^ until the desired extension was achieved. The stretch ratio (SR) was defined as the ratio of the final (stretched) length to the initial value (Figure S1). After wet-stretching, the filaments were dried in a vacuum oven at 120 °C and fixed under tension to avoid shrinkage during solvent evaporation. Filaments were produced at three different stretching ratios, denoted as DA-CMC/CNT@0, DA-CMC/CNT@50, and DA-CMC/CNT@100.

### Characterization

The morphology of CNT and DA-CMC/CNT was accessed by AFM imaging (Asylum Research Bio-MFP 3D, Oxford Instruments). The symmetrical interactions (between CNT and CNT as well as that between DA-CMC/CNT and DA-CMC/CNT) were measured by AFM’s quantitative image (QI) mode (tip model AC160TS, 9 ± 2 nm and AFM scanning resolution of 1024 × 1024). The optical birefringence of DA-CMC/CNT suspensions was observed by polarized optical microscopy (BX51 Olympus, Japan). The ζ-potential of the suspensions with pH = 7.4 was measured during 28 days using a Zetasizer (Nano ZS, Malvern) (with measurement every 7 days). The viscosity and yield stress of suspensions were measured using a rheometer (MAR S60, Thermo Fisher Scientific, Germany) operated at 25 °C. The surface and diameter of the fabricated filaments were measured by optical microscopy and SEM (S9800, Hitachi, Japan) operated at an acceleration voltage of 5.0 kV. The chemical groups and the state of DA/CMC-CNT filaments were analyzed by XPS (AXIS Ultra DLD, Shimadzu, U.K.) using a monochromatic Al Kα X-ray source. WAXS measurements were carried out using a NanoSTAR (Bruker-AXS Germany), with filaments placed perpendicular to the beam, making the X-ray radiation to pass vertically through the filaments. The electrical resistance of all samples was tested using a bench-type multimeter (UT-803, UNI-T, China) and recorded simultaneously (UT-803 Interface Progam_Ver: 0.1). *I*–*V* curves of the samples were obtained by an electrochemical workstation (CH Instruments Inc.) operating in the range from −0.5 to 0.5 V. The rheology measurements were carried out on DA-CMC/CNT suspensions using a rheometer (MCR 302, Anton Paar, Germany) operated at room temperature with a 25 mm diameter parallel plate set up with a truncation gap of 1 mm and using frequency measurement under 50% strain.

### Mechanical Properties

The tensile strength of the filaments was measured with a universal testing machine (SANS, CMT4202, China) operated at 10 mm min^–1^. Cyclic loading–unloading tests were used to test the strength of the composite interfaces in DA-CMC/CNT@100, DA-CMC/CNT@50, and DA-CMC/CNT@0, following a stress set in the range of 50–400, 50–200, and 50–100 MPa, respectively. The speed and cycle index were 10 mm min^–1^ and 1000. To quantitatively analyze the effects of 1000 cycles of axial stretching, the normalized variation of elastic modulus *Y* and strain *D* were employed
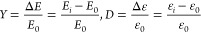
where *E*_0_ and ε_0_ are the initial elastic modulus and strain; *E_i_* and ε*_i_* are the elastic modulus and strain of the fibers undergoing the *i*th time loading–unloading cycle (*i* = 1, 10, 100, and 1000).

### Molecular Dynamics Simulations

All molecular dynamics simulations were performed by utilizing GROMACS package,^34^ version 2020.4. The carbon nanotubes used in our experiments were modeled using the GAFF force field.^35^ The electrostatic charges of DA-CMC and functionalized CNT were computed based on the MK-RESP (Merz-Kollman Restrained Electrostatic Potential) methodology.^36^ To obtain the RESP charge, single-point calculations were performed at the B3LYP/Def2-SVP level^37^ using Orca 4.2.1;^38^ RESP fitting of charges was performed using Multiwfn.^39^ The charges of functionalized CNTs were assigned considering the electrostatic charges of the carbon atoms near the carboxyl groups, handled according to the results RESP fitting, while the electrostatic charges of other carbon atoms were regarded as zero. Subsequently, the structure, including two CNTs with different skew angles and wrapped by DA-CMC, was obtained after undergoing NPT ensemble at 300 K and a constant pressure of 1 bar to equilibrate the samples. Then, the structure was quenched at ambient pressure to 0 K and relaxed to arrive at an equilibrium configuration. The shear stress was applied at the bottom and top of the structure at the speed of 0.001 nm ps^–1^, and each sample was tested at least 8 times to obtain an average. The energy was analyzed using gmx energy tool. The π–π stacking angle and distance were calculated through VMD.^40^ The atomic shear strain and two-dimensional contour of shear strain were determined utilizing OVITO.^41^

## Results and Discussion

DA-CMC/CNT filaments were synthesized by wet spinning followed by wet drawing, as shown in the schematic illustration of Figure 1a. The extrusion and flow effects contributed to the partial alignment of the building blocks; meanwhile, calcium ions present in the antisolvent (coagulant) neutralized the electrostatic charges of the DA-CMC and CNT system. After the formation of gelled filaments in the antisolvent, they were subjected to wet drawing, promoting further orientation in the axial direction, improving filament’s mechanical strength and conductivity.^42^ It is reasonable to assume that the motion of the gelled filament was likely governed by the viscoelastic properties of the initial suspension, which allowed for the building blocks to unidirectionally slip rather than fracture, which otherwise would occur in the presence of voids or defects. Figure S2 displays gelled filaments stretched over 3.17 times, leading to the expectation of orientational assembly by wet drawing. Herein, we developed CNT-based gel filaments following a smooth liquid–solid transition, enabling deformation and alignment of DA-CMC and CNT.

Polarized optical microscopy (Figure 1b) showed the presence of liquid crystal domains of DA-CMC/CNT with schlieren textures, revealing the local orientation of the suspension within disclination boundaries.^43^Figure 1c shows a shear stress–strain profile measured for DA-CMC/CNT suspensions, indicating a yield stress region, which was selected for wet drawing, e.g., to induce partial molecular slippage and unfolding, which are effective to avoid catastrophic fracture and excessive sliding. Figure S3 shows the steady response of the hydrogels to oscillation sweep (see their consistency in Figure S4a), with a storage modulus of 100 Pa under an applied strain of 50 and 100%, implying a high strength development upon drying.^44^ The viscosity of neat CNT suspensions (11.3 Pa·s) was lower than that of the DA-CMC/CNT suspensions (95 and 256 Pa·s, respectively, Figure S4b), which can be attributed to the enhanced aqueous dispersion in the presence of DA-CMC (see also the shear stress of the suspensions under strain sweeps, Figure S4c). The yield stress of the DA-CMC/CNT-50 suspension (15 Pa) was higher than that of neat CNT (7.9 Pa) and DA-CMC/CNT-20 (8.99 Pa) suspensions (Figure S5b), e.g., the former needed higher energy to break the clusters and to initiate the reorganization of the components.

Atomic force microscopy (Figure S5a) indicated a CNT diameter of ∼1.5 nm. As a comparison, the diameter of DA-CMC wrapped on CNT was ∼10 nm, as a consequence of the interfacial interaction between the components,^33^ and entropy-driven self-assembly (Figure S6). The ζ-potential of the suspensions measured after 28 days was −40 mV, showing a colloidally stable system suitable for wet spinning (Figure S5c).

The two-dimensional free energy results calculated by meta-eABF approach are displayed in Figure S6a, in the left corner of the map, with the highest free energy. As shown in Figure S6b, the free energy map demonstrates that DA-CMC wrapped around CNT in the aqueous cavity with minimum free energy. The entropy changes indicated that the dispersion of CNT in aqueous DA-CMC is a spontaneous process. DA-CMC and CNT wrapping, as well as the clusters of nonbonded interactions, contributing to dissipating energy during the process, increased both the viscosity and yield stress. These results, indicating a transition from a colloidal suspension to macroscopic filaments, allowed us to anticipate a highly aligned structure with a high cohesion between the building blocks, a subject of further discussion.

**Figure 2:**
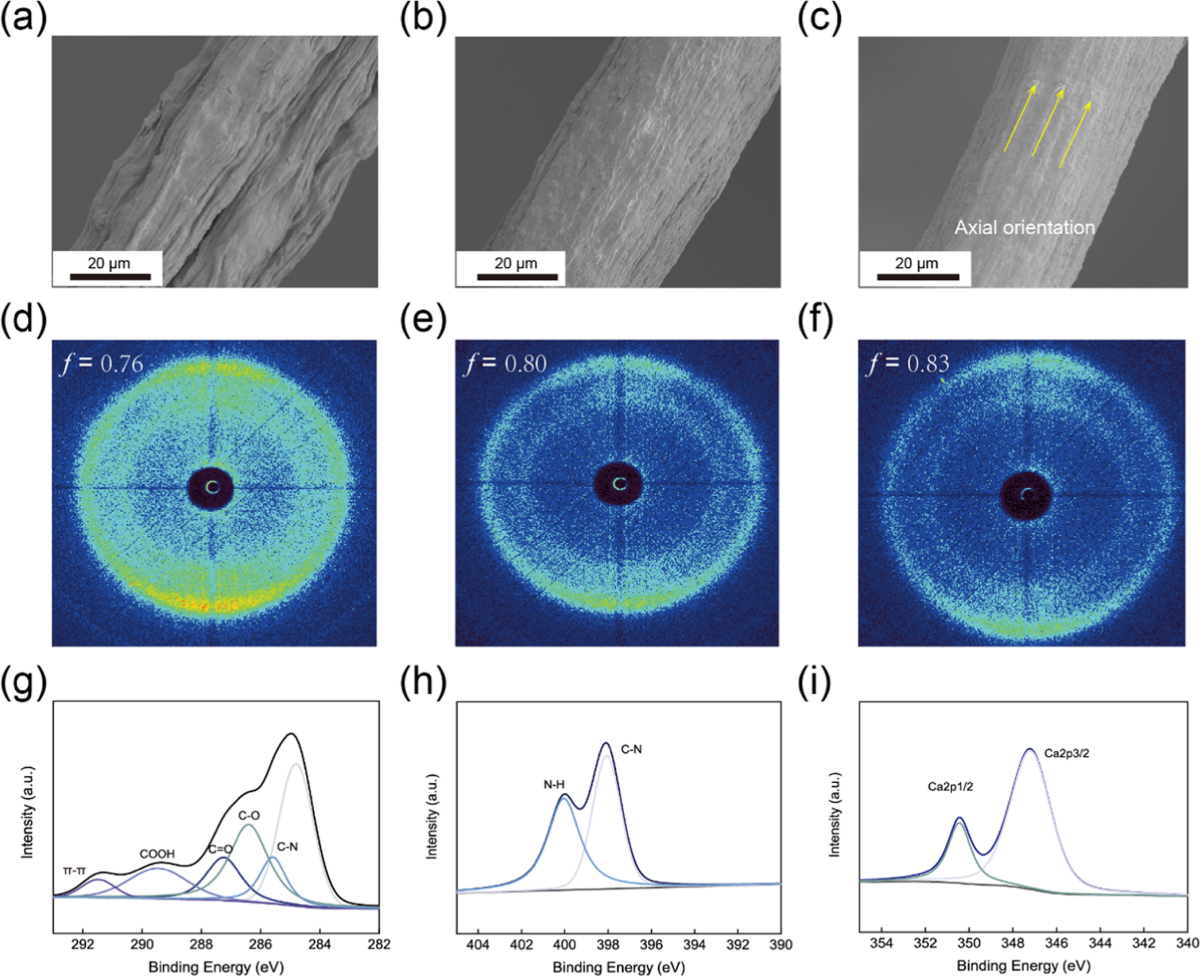
Structural analysis applied to DA-CMC/CNT filaments. SEM images of a composite filament with different stretching ratios (SRs): (a) DA-CMC/CNT@0, (b) DA-CMC/CNT@50, and (c) DA-CMC/CNT@100. WAXS patterns of the composite filament of (d) DA-CMC/CNT@0, (e) DA-CMC/CNT@50, and (f) DA-CMC/CNT@100. XPS spectra of (g) C 1s, (h) N 1s, and (i) Ca 2p in DA-CMC/CNT@100.

In the absence of wet drawing, rough hybrid filament surfaces were obtained (Figure 2a) mainly due to the Brownian diffusion of CNT.^45^ In comparison, the filaments obtained after wet drawing displayed smooth surfaces (Figure 2b,c). The diameter of the composite filaments obtained at given stretching ratio (SR) varied from 51 to 41 μm, while the density was between 1.32 and 1.54 g cm^–3^, e.g., a denser structure developed upon wet stretching (Figure S7, Table S1). The alignment of the building blocks was experimentally confirmed by the orientation index (*f*) obtained from the characteristic full width at half-maximum of the azimuthal profiles obtained by wide-angle X-ray scattering (WAXS) (Figures 2d–f and S8). In the absence of wet drawing, DA-CMC/CNT@0 exhibited a lower alignment (*f* = 0.76) compared to DA-CMC/CNT@50 (*f* = 0.8) and DA-CMC/CNT@100 (*f* = 0.83). Hence, a higher stretching ratio produced a more unidirectional and denser arrangement of the constituents and indicated that DA-CMC enhanced CNT axial organization.

X-ray photoelectron spectroscopy (XPS) was used to investigate the chemical composition affecting the interactions between DA-CMC and CNT. As shown in Figure 2g, the peak attributed to π–π interactions at 291.5 eV appears in the C 1s spectrum. In our previous study,^45^ we confirmed the conjugation of DA with CMC using XPS. We suggested that the C 1s spectra tracked with the pi-pi interactions of the systems containing CNT, showing strong sp^2^ carbon–carbon bonds and hexagonal network structures. The latter denote the interaction between catechol in DA-CMCs and the carbon rings of CNT. We further note the UV–vis spectra (Figure S9) comparing the DA-CMC and DA-CMC/CNT, indicating the characteristic peaks at 230 and 300 nm, attributed to the π–π transition of C=C and the n–pi transition of carboxyl functional groups. The XPS peak of C–N bonding in C 1s and N 1s spectra confirmed the introduction of DA-CMC. Two peaks, corresponding to Ca 2p_1/2_ and Ca 2p_3/2_, demonstrated that the addition of Ca^2+^ generated coordinated bonds with DA-CMCs as well as the carboxyl groups in CNT. Hence, as is the case of other polymers, Ca^2+^ acted as a crosslinker, which is a topic that deserves attention on its own but is not discussed in the present study for brevity. For instance, the role of polymer architecture, vicinal water, and hydrogen bonding on the mechanisms of counterion binding and interactions are quite complex but critical for a thorough understanding of the system.^46^

**Figure 3:**
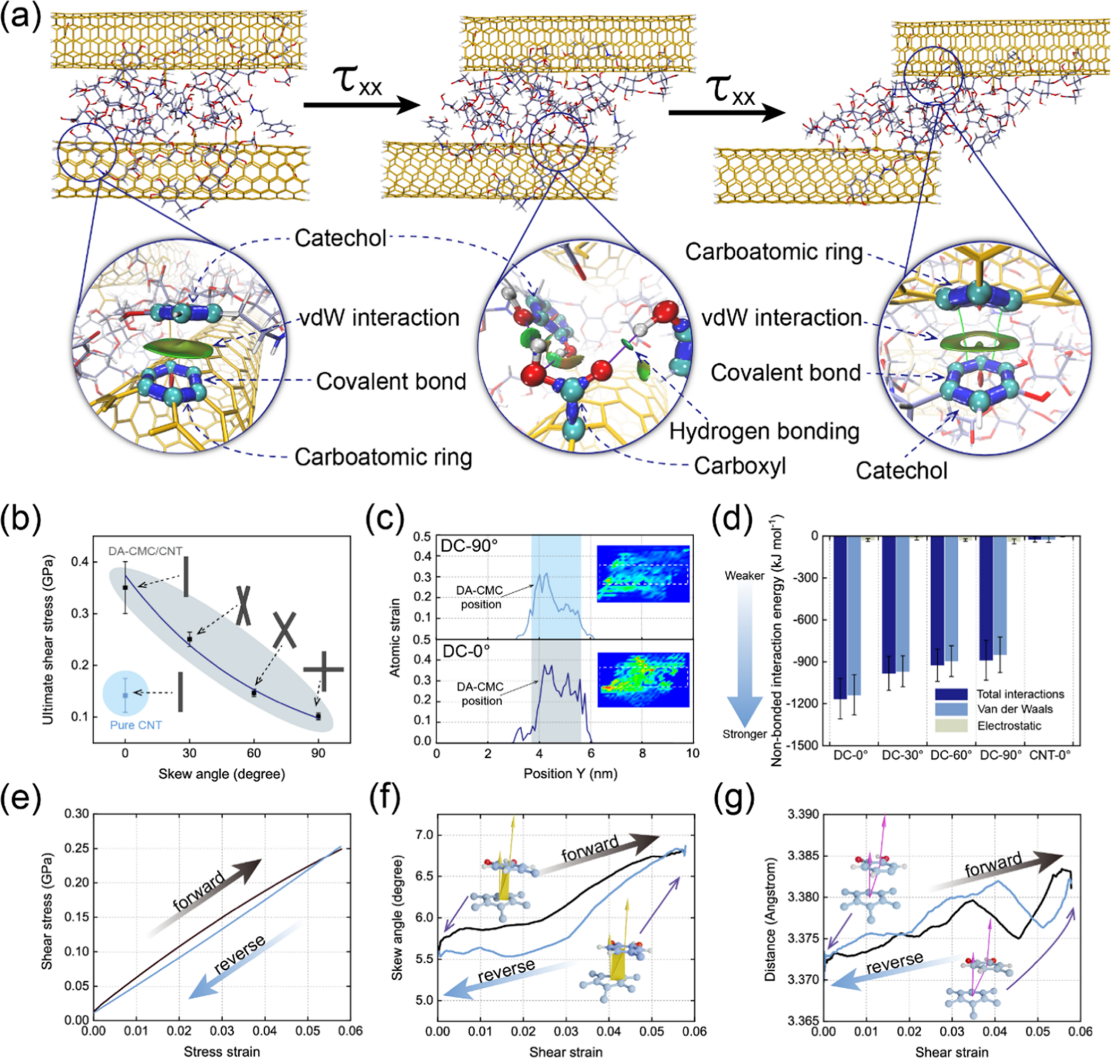
Molecular dynamics simulation analyses of interfaces between DA-CMC and CNT under different orientations. (a) Schematic illustration of a DA-CMC/CNT structure undergoing interfacial shear stress with parallel CNT. Partially enlarged images are included to show the interactions between DA-CMC and CNT, including vdW forces as well as hydrogen bonding, as noted. (b) The ultimate shear stress of DA-CMC/CNT structures corresponding to different skew angles (0, 30, 60, and 90°) between the CNTs, designated as DC-0°, DC-30°, DC-60°, and DC-90° (see details in Figure S10, corresponding to each skew angle). (c) Atomic strain comparison for DC-0° and DC-90° configurations. (d) The nonbonded interaction of DC-0°, DC-30°, DC-60°, DC-90°, and neat CNT-0° structures bearing shear stress. (e) The stress–strain curve of DC-0° undergoing loading–unloading shear stress. (f) The skew angle variation and the (g) distance variation between a catechol in DA-CMC and a carboatomic ring in CNT when the DC-0° structure is under cyclic shear stress.

Based on the experimental evidence discussed so far, we focus our attention on studies conducted by molecular dynamics simulation aimed to gain further insights into the interactions and structural changes of the system. Structures comprising two nanotubes in each system were subjected to shear stress under different skew angles (0, 30, 60, and 90°, see modeling structures in Figure S10). Figure 3a displays the schematic diagram of the sliding process of DC-0° molecules, highlighting the π–π interaction and hydrogen bonding during the deformation. DA-CMCs underwent van der Waals (vdW) interactions with adjacent CNTs and included π–π and anion−π interactions.^33^ The magnified image depicts π–π stacking of catechol in DA-CMC and a carboatomic ring in CNT before and after deformation. They relate to the characteristic fracture and reformation under stress, which is in accordance with previous studies on hydrogen bonding of nanocellulose matrices.^47^ The carboxyl groups in CNT formed hydrogen bonding when the hydroxyl groups in DA-CMC moved to their vicinity, suggesting that the rearrangement of building blocks or the disentangling of the original networks facilitated a strong interfacial structuring, Figure S11, where the DC-0° structure reached an ultimate shear stress of 0.35 GPa after the sliding of the building blocks. Figure 3b displays the ultimate shear stress of structures with different orientation angles. When the skew angle of CNT was 0 (DC-0°), the shear stress was almost 2.7 times that of the neat CNT structure. At an increased skew angle (60°, DC-60°), the shear stress of the DA-CMC structure (0.15 GPa) was higher than that of CNT in the parallel condition (0.14 GPa), which is due to the molecular interlocking in the form of molecular zip-up that is expected with the introduction of DA-CMCs.^48^ The atomic strain can be spread homogeneously in the structure, in the deformation path, where the overall constituent strain at DC-0° was higher than 0.3 (Figure 3c). In contrast, half of the component strain in DC-90° was below 0.3, implying local stress and strain concentration, which negatively influenced the load transfer and led to local crack propagation. The nonbonded interaction energy generated during the process demonstrated that unidirectional building blocks exhibit stronger interactions, which means that wet drawing induced further aligned structures (DA-CMC/CNT) in the filament and increased the resistance to interfacial shear stress. As displayed in Figure 3d, the highly oriented conformation (DC-0°) exhibited the highest nonbonded results, reaching −1170 ± 150 kJ mol^–1^ (note that for DC-90°, this value was −900 ± 143 kJ mol^–1^). By comparison, the nonbonded interaction generated between neat carbon nanotubes was −50 kJ mol^–1^, further demonstrating that DA-CMC improved the mechanical strength for DA-CMC/CNT composite filaments.

To explore the intermolecular stick and slip effects, we observed the molecular behavior in the region of yield shear stress under cyclic loading–unloading. Figure 3e shows a reversible deformation under complete shear stress at DC-0°, demonstrating that the interlocking through nonbonded interactions can sustain interfaces instead of relative slippage at low strain. The distance and the relative angle between catechol in DA-CMC and the carboatomic ring in CNT can attain recovery, namely, the skew angle between these groups was recovered between ∼6.75 and ∼5.5°, and the distance exhibited a ∼0.012 Å fluctuation (Figure 3f,g). However, for the DC-30° case, the shear stress–strain curve showed no recovery to the original state after deformation (Figure S12), and the structure displayed a ∼0.5% residual strain. Compared with the DC-0° structure, the irreversible deformation can be attributed to the release of energy during plastic deformation.^49^

From the perspective of molecular structuring, there is an indication that the recovery phenomenon is limited, given the irreversible changes in the skew angle and distance between catechol and carboatomic rings (Figure S13), suggesting that pre-existing defects can lead to the dislocation between atoms. The irreversible changes of the skew angle and distance demonstrated that the catechol groups in DA-CMC cannot remain attached to CNT, leading to irreversible deformations. In other words, neighboring groups can adhere to each other through intermolecular torsion and molecularly unfold in the yield shear stress region of the structure.

Prior to the continuous slippage of vicinal building blocks, the stretch of molecular chains is critical in determining the strength of the configuration. As shown in Figure S14, the dihedral angle (N–C–C–C) underwent a reversible variation for DC-0°, confirming that the catechol groups maintain π–π stacking with CNT under tension and in the form of structural torsion. The dihedral angle for DC-30° cannot regain the initial state, demonstrating a dislocation slippage between DA-CMC and CNT. Thus, for a higher axial orientation, the strong wrapping around CNT enhances the strength and stability of the structure via nonbonded interactions and enables the recovery of torsion, resisting interfacial shear stress. On the macroscopic scale, less-ordered structures cannot bear high shear stress and are subjected to crack propagation between neighboring components at low strain, leading to stochastic fracture.^50,51^

**Figure 4:**
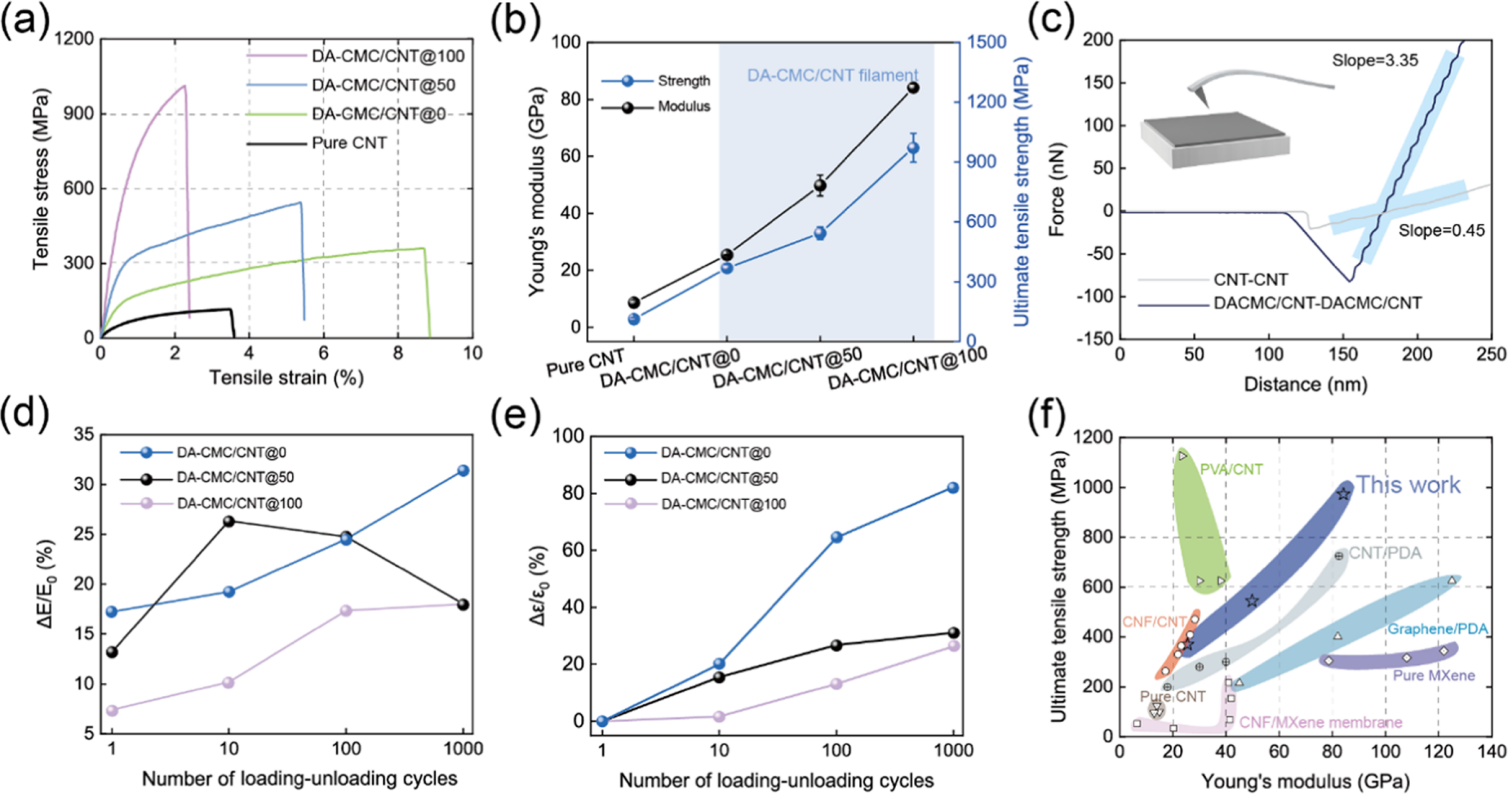
Mechanical properties of DA-CMC/CNT filaments obtained at different stretching ratios (SRs). (a) The stress–strain curves of DA-CMC/CNT and CNT filaments. (b) Young’s modulus and ultimate tensile strength of hybrid and CNT filaments obtained at different SRs. (c) Magnified *F*–*D* curves from pull-off tests for the interaction between CNT Si tip and CNT surface as well as DA-CMC/CNT Si tip and DA/CMC/CNT surface. (d) Normalized Young’s modulus of composite filaments with different SRs withstanding 1000 loading–unloading cycles. (e) The normalized strain (ε) of composite filaments obtained at different SRs undergoing 1000 loading–unloading cycles. (f) Ashby plot of Young’s modulus versus ultimate tensile strength including the results of CNT, Mxene, graphene, cellulose nanofibrils (CNF), and their composite filaments (see Table S2).

Following the discussion about the role of interfacial behavior at the molecular scale, Figure 4a displays the experimental stress–strain curves of composite and CNT filaments obtained at different SRs (Table S1). The introduction of DA-CMC improved both toughness and strength. The filaments produced in the absence of drawing (DA-CMC/CNT@0) exhibited a tensile strength of 369 MPa and a toughness of 24.7 MJ m^–3^. In comparison, neat CNT fibers showed 114 MPa and 3.1 MJ m^–3^, respectively (Figure S15). The elastic modulus and tensile strength increased after wet drawing, with Young’s modulus going from 25.5 to 84.1 GPa and tensile strength from 369 to 972 MPa (Figure 4b). This strength improvement can be ascribed to the formation of more isotropic structures affecting the axial properties and enhancing the strength between neighboring building blocks as a consequence of an ordered packing. In units typical of textile materials, Figure S16 refers to a specific strength of 0.65 N tex^–1^ for DA-CMC/CNT@100, exceeding the mechanical performance of nanocellulose fibers (∼0.29 N tex^–1^) and polyester-polyolefin fiber yarns (0.59 N tex^–1^).^31,52^

We speculate that hydrogen bonding and van der Waals (vdW) interactions between the DA-CMC matrix and the CNT allowed load transfer within the materials rather than relative weak interactions that lead to slippage of the building blocks at low strain.^18,53^Figures 4c and S17 show AFM force–distance curves for CNT/CNT and DA-CMC/CNT. The pull-off force for the latter is 82 nN, which is almost 4 times larger than that of CNT/CNT (21 nN). The enhanced pull-off force is primarily caused by the strong adhesion between DA-CMC and CNT as well as the strong interaction within the matrix itself through attractive vdW and hydrogen bonding.^14^ The slope of the strength profile of DA-CMC/CNT is 3.35, approximately 7.4 times higher than that of CNT, indicating that the presence of DA-CMC stiffens the interfaces.

Experiments involving cyclic loading–unloading were conducted to further investigate the influence of orientation on the interfacial strength (see stress–strain profiles after 1000 cycles in Figure S18). The strain of DA-CMC/CNT@100 was 0.61% at a stress of 400 MPa. Also, Figure S18b,c reveals 1000 cyclic strains with a maximum stress of 200 and 100 MPa for DA-CMC/CNT@50 and DA-CMC/CNT@0, respectively. As a result of the plateau observed by the stress-hardening ability, the elastic modulus of DA-CMC/CNT@100 filaments underwent a limited increase with cycle numbers, 7.4, 10.2, 17.4, and 18.0%, respectively (see Figures 4d and S19 for SEM images and EDS maps of DA-CMC/CNT@100 filament’s cross section after 1000 loading–unloading cycles). DA-CMC/CNT@50 tended to a maximum increase in modulus (from 17 to 32%). The effect of strain hardening was likely due to local plastic deformation induced by the matrix network to slide for further alignment. In comparison, the elastic modulus variation of DA-CMC/CNT@0 filaments started to decrease after 10 loading cycles, which can be attributed to relatively weak interfaces. Indeed, strain can boost the strength of filaments; however, an excessive stress can lead to the overall slippage of the matrix network, especially in materials with weak interfaces.

The normalized strain variation (Δε/ε_0_) shows that the filaments with higher orientation sustained reversible deformation (Figure 4e). The maximum expansion rate under the axial strain of DA-CMC/CNT@0 reached ∼82%, which is ∼2.7 and ∼3 times that of DA-CMC/CNT@50 and DA-CMC/CNT@100, respectively. The filaments produced in the absence of wet drawing exhibited an increased rate of irreversible deformation, which is ascribed to lower internal friction of the entangled network structure. The interfaces in DA-CMC/CNT@100 filaments prevent early fatigue in bulk materials, usually caused by local stress concentration in the matrix. A higher orientation structure facilitated interfaces with improved ability to bear stress, preventing debonding before material failure. On the other hand, the interfaces cannot withstand local stress concentration, leading to the yielding stress in the early stages of strain.

The enhanced interactions between components and the strain-induced reorientation of molecular chains demonstrate a stiffening mechanism, which has been applied in the fabrication of nanocarbon,^42,54^ biomacromolecular^55,56^ and polymeric^57^ fibers. However, the aligned structure can accumulate substantial stress within the materials, which can lead to a brittle failure. For example, well-ordered MXene fibers achieved a modulus of 122 GPa, while their strain decreased from 0.4 to 0.2%.^58^ In comparison, our fabricated fibers exhibited a suitable toughness (14.2 MJ m^–3^, SR = 100%, Figure 4f), which can be attributed to a wide distribution of nonbonded interaction between DA-CMCs and CNT.^59^ Composite materials produced with CNT fibers mixed with poly(vinyl alcohol) (PVA) displayed excellent failure strain (up to ∼12.5%), whereas the elastic modulus reached a value of 40 GPa,^60^ a value that is almost half compared to that of the DA-CMC/CNT@100 system. The performance of the latter is explained by the effects of coordinate bonding (Ca^2+^) and long-range π–π interactions, resulting in energy dissipation that impedes interfacial fracture^61,62^ (see Figure S19). Our previous studies demonstrated that cellulose nanofibrils (CNF)/CNT filament presented a tensile strength of 472 MPa and modulus of 28 GPa.^23^ By contrast, the tensile strength and Young’s modulus of the filaments in this work were ∼2 and ∼3 times higher, as a result of the effect of structural alignment, the coagulant type,^44^ and the wet drawing.

Based on the excellent mechanical properties of the aligned DA-CMC/CNT filaments, we next explore their applications as conductive systems. Following the alignment of CNT structures, the composite filaments demonstrated a suitable electrical conductivity; Figure S20. The conductivity of DA-CMC/CNT@100 reached 243 S cm^–1^, which is nearly 2.3 times that of DA-CMC/CNT@0. Both the high colloidal stability of the CNT and DA-CMC and the alignment in the filament contributed to electrical percolation.^23,53^ The DA-CMC/CNT@100 produced a stable interface that withstood the applied load. Compared with metallic materials used for human–machine interfacing,^6,63^ the DA-CMC/CNT filaments presented a competitive Young’s modulus and toughness, offering the possibility for signal acquisition and transmission in textile materials.^64^ As an example, Figure S21a displays a setup that includes two filaments placed in a cross configuration and supporting a woodblock (10-g load, about 250 000 times the weight of the filaments). They were energized through 15 V DC power, and Figure S21b shows a negligible current variation during cyclic powering (on-and-off with a current maintained at around 7.8 mA). Figure S21c displays the voltage–current curves for three types of composite filaments, demonstrating stability under alternating voltage (AC).

The composite filaments displayed an outstanding performance for electrothermal imaging. Figure S21d,e shows the temperature variation for DA-CMC/CNT filaments shaped like an “S” under a stepwise voltage, from 3 to 15 V. The inset image displays the temperature contours of the composite filaments. The results showed no signs of short-circuiting, denoting a relatively steady migration of electrons within the structure. Meanwhile, to examine the stability and electrothermal performance, we applied a cyclic power (on-and-off) with filaments reaching a saturation temperature (∼41.5 °C) within 50 s. They cooled down naturally to room temperature after 6 cycles (Figure S21f). The filaments assembled in other shapes did not exhibit any significant fluctuation in Joule heating, indicating excellent electrothermal stability.

Finally, we demonstrate the application of DA-CMC/CNT filaments as a breathing sensor (Figure S21g). To improve the sensitivity to electrical resistance, we combined the filaments into yarns and coated them with PVA (used to hold them together). The yarns were sewn into a surgical mask and connected with wires through conductive silver adhesives (Figure S21h). Given the carboxyl and hydroxyl groups in CNT and DA-CMC, the filaments swelled when they were exposed to high humidity, contributing to an increased electrical resistance. Figure S21i displays the normalized resistance variation during use: the yarn exhibited a reduction in electrical resistance since exhalation induced a strain in the conductive yarn, leading to an increased filament resistance. In another experiment, under the effect of sound vibrations (human voice), the recorded signals displayed four peaks, given filament deformation. Notably, the peaks tended to decline after several cycles, which can be ascribed to the water molecules absorbed on the surface of CNT, partially blocking electron transfer.^65^

## Conclusions

In summary, using molecular dynamics simulation, we explain the fundamental reasons for the enhancement of the interfacial interactions between the building blocks in CNT-based filaments, leading to alignment and improved strength and stiffness. We demonstrate that the dynamic loading/unloading of aligned structures (and the corresponding interfaces) support reversible distortion, remarkably gaining reversible deformation. The nature of DA-CMC binding and interactions with CNT, followed by strain-induced wet spinning, leads to filaments with a remarkable strength (up to 972 MPa, Young’s modulus 84 GPa). Such effect is not only relevant to the mechanical performance but also has an impact on other properties, such as electrical conductivity (241 S cm^–1^), making the system suitable for applications in sensing (breathing) and for electrothermal imaging. Overall, theoretical and experimental evidence show that the axial orientation of DA-CMC/CNT in the filaments facilitates interfacial interactions, enhances stress resistance, and prevents early fracture or yielding. Such results are also of relevance to applications involving electrothermal activity.

## Supporting Information Available

Schematic diagram of the process of DA-CMC/CNT filament stretching; the stretchability of filaments in the wet state; the storage and loss modulus of DA-CMC/CNT suspension with CaCl_2_ solution using strain sweep measurement; the optical photographs of filaments with different SRs and neat CNT filaments; details for molecular dynamics simulation, characterization of filaments, including stress–strain curves, electrical and electrothermal applications; and the table of mechanical properties and data used in the construction of the Ashby plot in Figure 4f (PDF)
Showing filament stretching (MP4)
