# Supplementary material for: Mechanisms of Strain-Induced Interfacial Strengthening of Wet-Spun Filaments
Source: ACS Appl Mater Interfaces. 2022 Mar 30;14(14):16809–19. doi: 10.1021/acsami.1c25227 (PMC9011349; doi:10.1021/acsami.1c25227)
Supplement: Supplementary file 1 — am1c25227_si_001.pdf [file am1c25227_si_001.pdf]

Supporting Information

Mechanisms of Strain-Induced Interfacial Strengthening of Wet-spun Filaments

Tianyu Guo[1,2], Zhangmin Wan[2], Yan Yu[2], Hui Chen[1], Zhifeng Wang[4], Dagang Li[3], Junlong Song[1], Orlando J. Rojas[2,5], Yongcan Jin[1,*]*

[1]Jiangsu Co-Innovation Center of Efficient Processing and Utilization of Forest Resources, and Jiangsu Provincial Key Lab of Pulp and Paper Science and Technology, Nanjing Forestry University, Nanjing 210037, PR China;

[2]Bioproducts Institute, Department of Chemical and Biological Engineering, Department of Chemistry and Department of Wood Science, The University of British Columbia, 2360 East Mall, Vancouver, BC V6T 1Z3, Canada;

[3]College of Material Science and Engineering, Nanjing Forestry University, Nanjing 210037, PR China;

[4]Testing Center, Yangzhou University, 48# Wenhui East Road, Yangzhou 225002, PR China;

[5]Department of Bioproducts and Biosystems, School of Chemical Engineering, Aalto University, P. O. Box 16300, FI-00076 AALTO, Finland.

*Corresponding authors: (JY) jinyongcan@njfu.edu.cn; Tel: +86 (25) 85428569; (OJR) orlando.rojas@ubc.ca, Tel: +1-6048223457

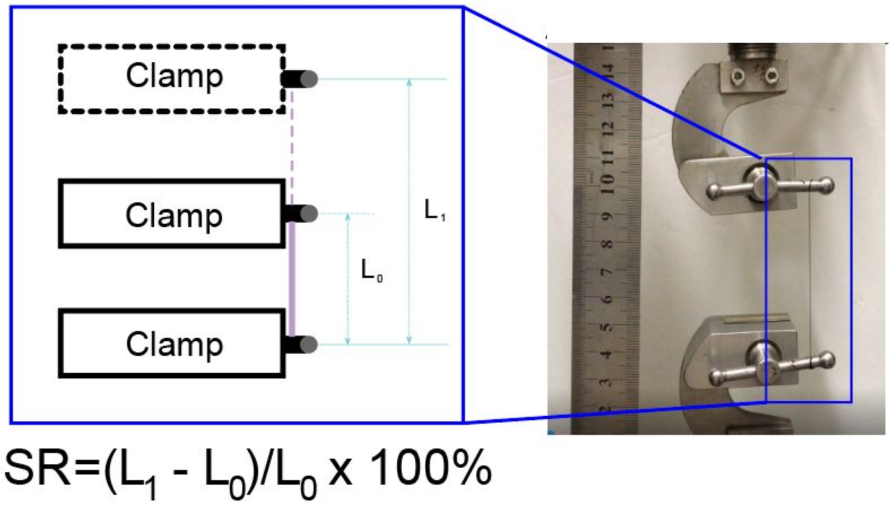

Figure S1 Schematic diagram illustrating the process of stretching DA-CMC/CNT filaments. The gel threads were fixed on the both ends of the clamp. Then the gel threads were stretched at a strain rate of 15 mm min^{-1}. The semi-finished filaments were placed in a vacuum oven at 120 °C for 24 hours to dry, fixed under tension to avoid shrinking during the process of drying.

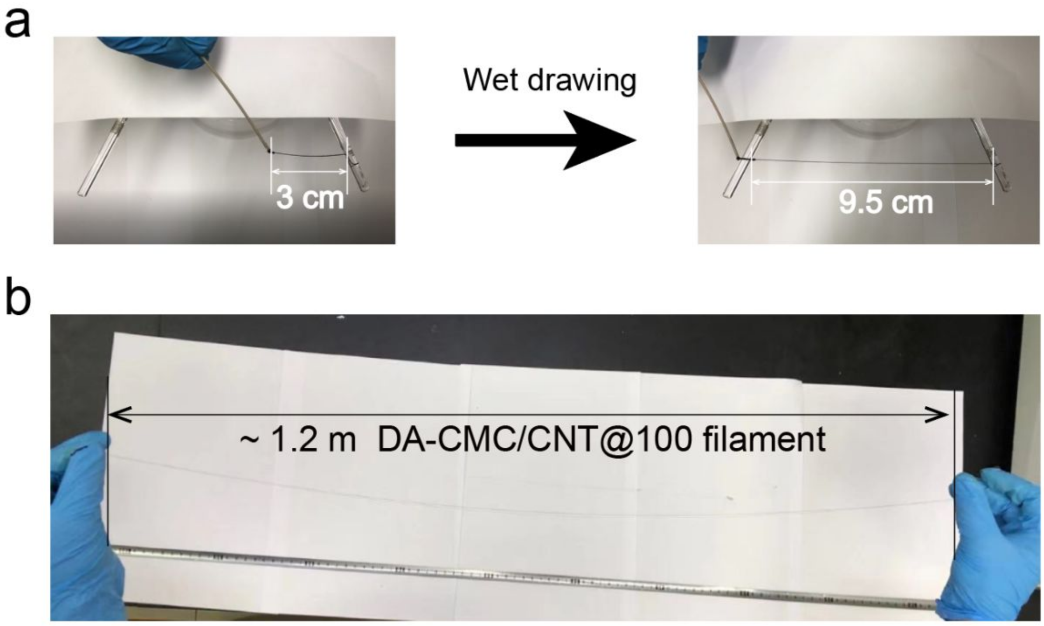

Figure S2. Stretchability of DA-CMC/CNT filaments in the wet state. (a) The gels threads (3 cm to 9.5 cm) form under tension. (b) 1.2-m length DA-CMC/CNT filament using a stretching ratio of 100%. In (a), the gel fibers can be extended ~ 3.2 times of their original length, from 3 cm to 9.5 cm after wet drawing, showing ductility. Figure (b) displays a ~1.2 m gel fiber. The gel fiber can be manipulated with no structural fracture, indicating the high mechanical strength of the composite filaments.

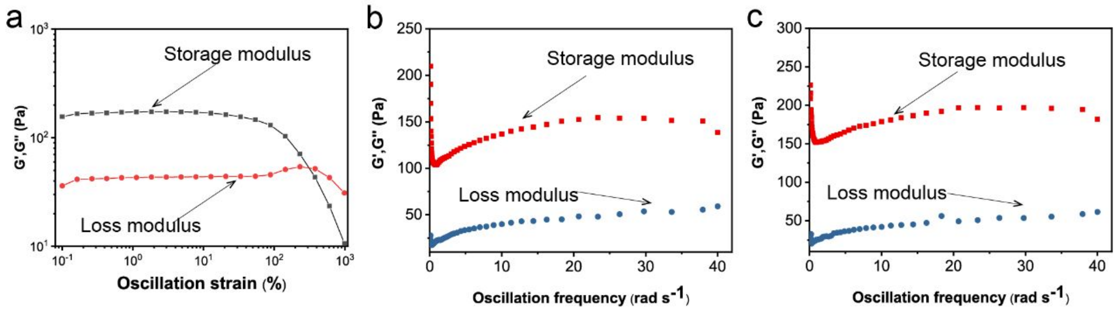

Figure S3. (a) The storage and loss modulus of DA-CMC/CNT suspension with $CaCl_2$ solution using strain sweep measurement. (b) The storage and loss modulus of DA-CMC/CNT suspension with $CaCl_2$ solution at 100% strain using frequency measurement. (c) The storage and loss modulus of DA-CMC/CNT-50 suspension with $CaCl_2$ solution at 50% strain using frequency measurement.

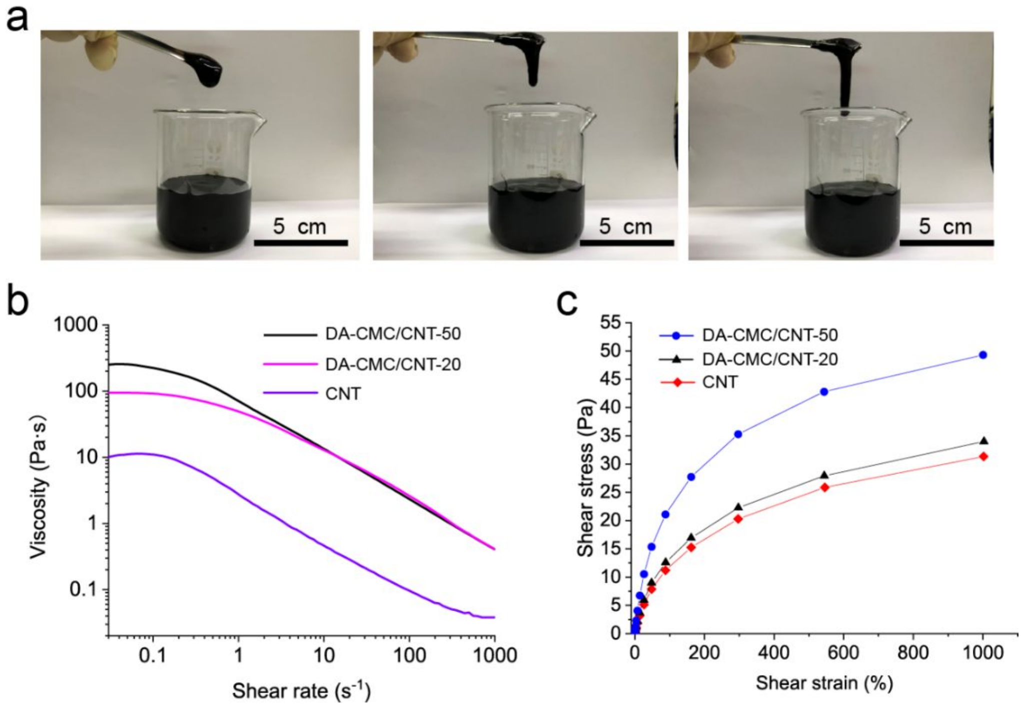

Figure S4. (a) Photographs of DA-CMC/CNT suspension (1:1). (b) The viscosity of composite and neat CNT suspensions. (c) The shear stress of composite and neat CNT suspensions using strain sweep measurement.

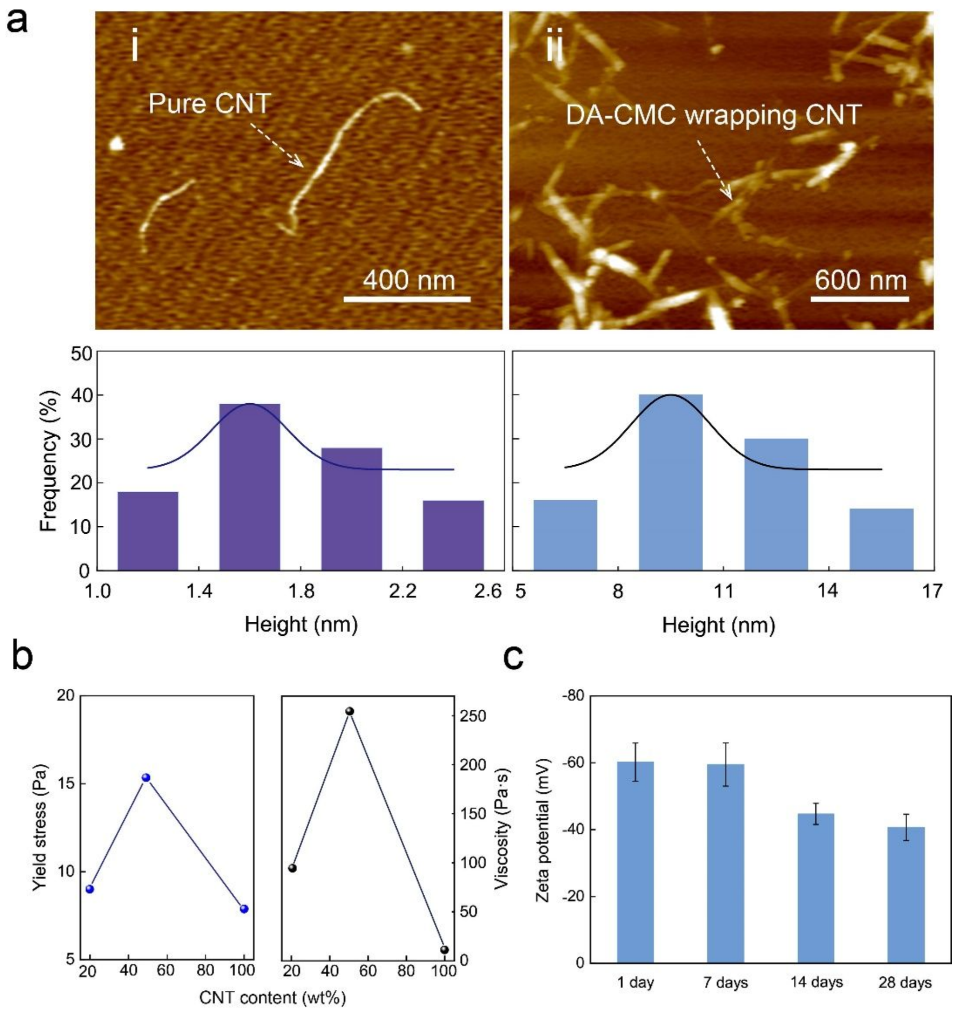

Figure S5 (a) AFM images of neat CNT and DA-CMC. In the latte case, polymer wrapping around CNT is shown. The height distribution is also included, which indicated important differences between the systems. (b) Yield strength and viscosity of the suspensions at given CNT content. (c) Zeta potential of the suspensions measured at different times.

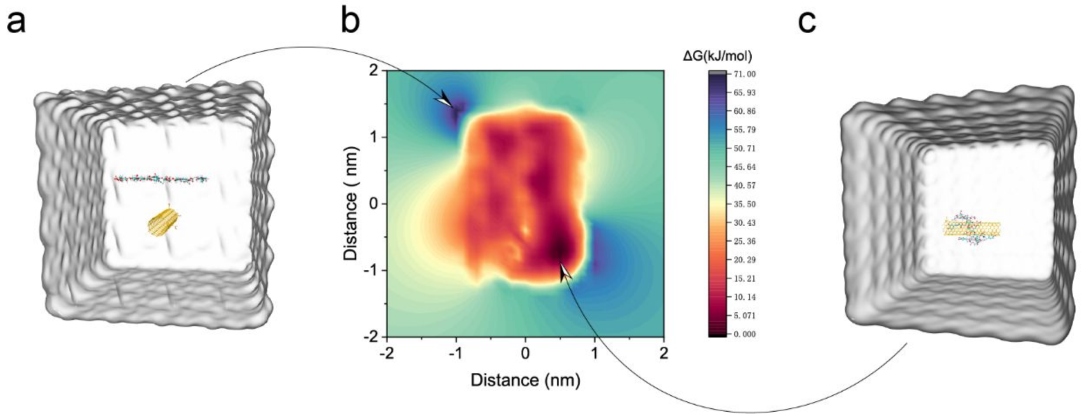

Figure S6. Free energy analysis of DA-CMC and CNT. (a) The configuration of DA-CMC and CNT before simulation. (b) Free energy surface as a function of the distance between a catechol in DA-CMC and the center of mass of CNT (x-axis) and the distance between the other catechol in DA-CMC and the center of mass of CNT (y-axis). (c) The configuration of DA-CMC wrapping CNT after 20 ns simulation. All the simulations described herein were conducted using Gromacs program,[1] version 2020.4, with GAFF force field [2] and SPC/E water model.[3] The simulation was performed in the NPT ensemble to obtain equilibrium configuration for 1 ns (the temperature was set at 300 K and the pressure at 1 bar). The simulation was carried in the NPT ensemble with the same parameters in the equilibrium simulation, where the cut-off of Lennard-Jones interactions and the real-space part of electrostatic interactions was 1.2 nm. Constraints were applied to hydrogen bonds using LINCS.[4] All the visualization of MD trajectories were performed with VMD, version 1.9.3.[5] Free-energy calculations were carried out using the meta-eABF method[6] through Colvars.[7] The transition of coordinates of the calculation was chosen as the distance between catechol in DA-CMC and CNT. The simulation of DA-CMC dispersing CNT was set to be 20 ns.

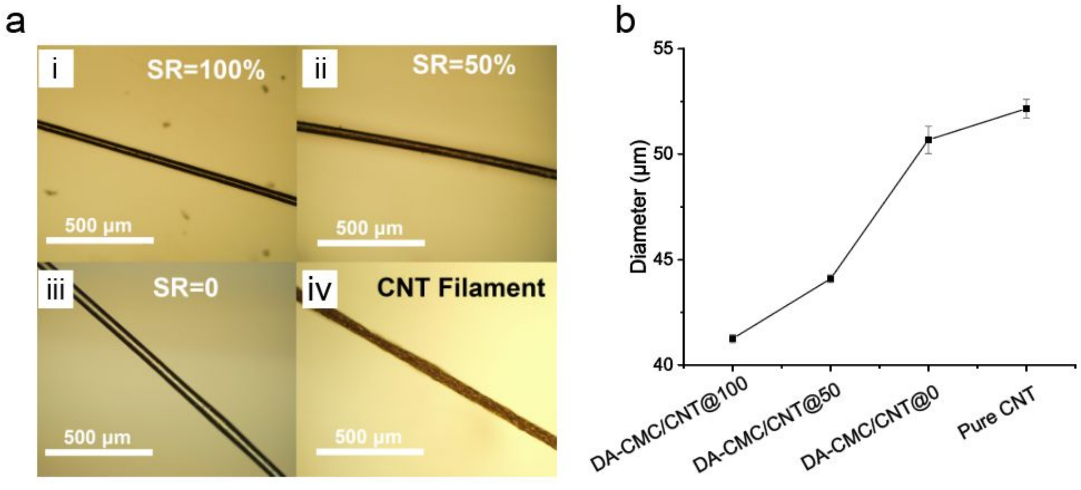

Figure S7. (a) Optical photographs of filaments with different SR and pure CNT filaments and the (b) diameters.

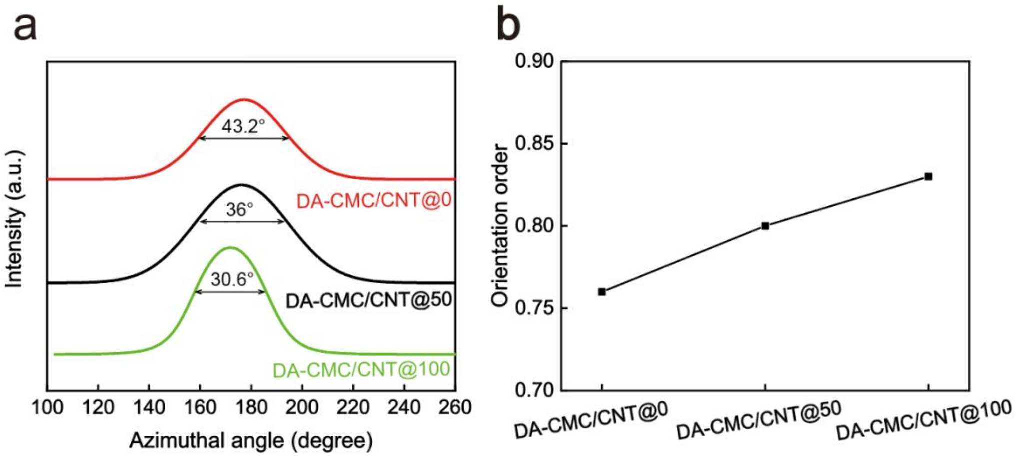

Figure S8. (a) The azimuthal angle profile of the DA-CMC/CNT filaments with different stretching ratios. (b) The orientation order calculated according to f=(180°-ΔΦ)/180°, where ΔΦ represents the full width at half maximum of the azimuthal scanned peaks.

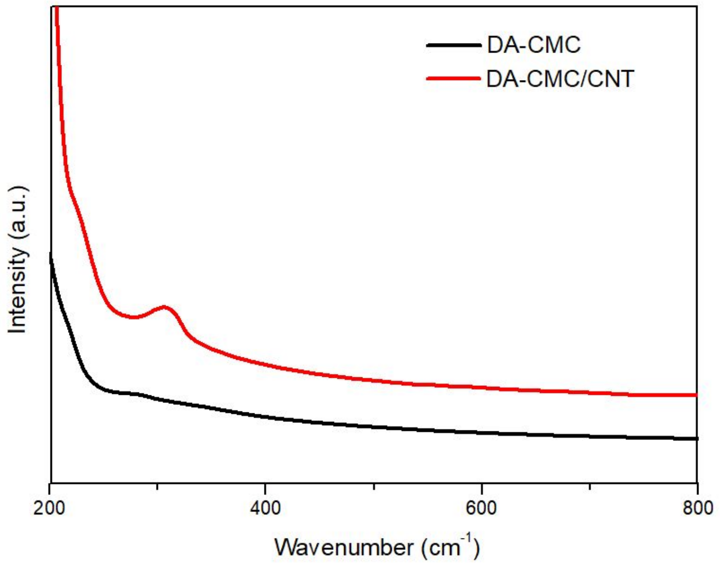

Figure S9. UV-vis absorption spectra of DA-CMC and DA-CMC/CNT. The spectra were recorded using a SHIMADZU UV-2600. Compared to DA-CMC, the UV-vis spectra of DA-CMC/CNT provides characteristic peaks at 230 and 300 nm, which are attributed to pi-pi transition of C=C and the n-pi transition of carboxyl functional groups. This further indicates the presence of pi-pi interactions in CNT.

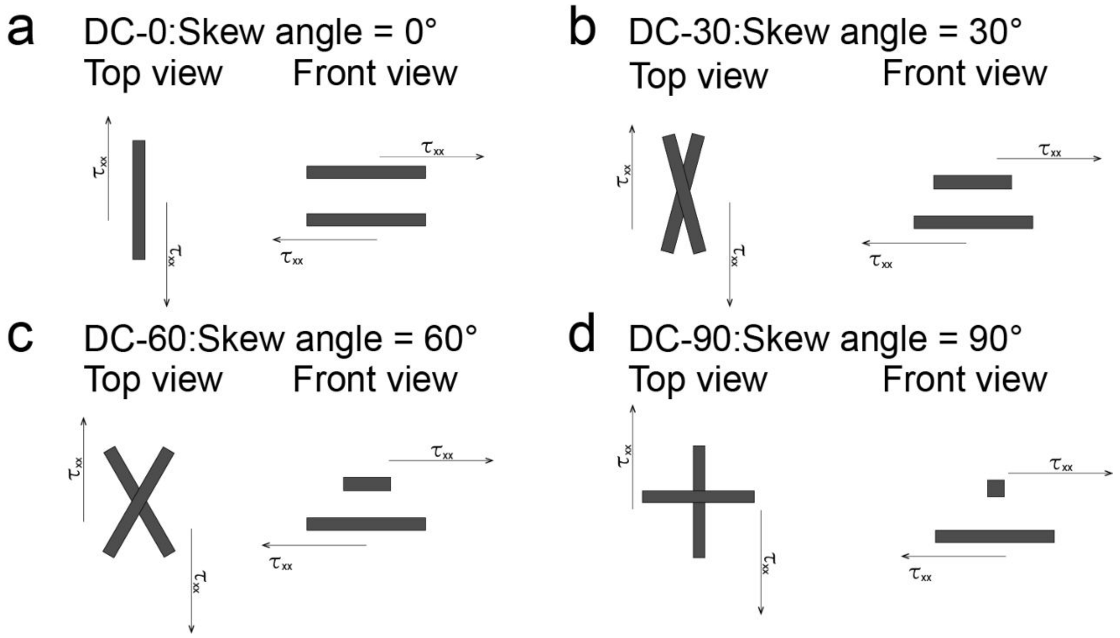

Figure S10. **Molecular dynamics simulation on the strength and structure of interfaces:** The schematic structure of CNT with different skew angles (only displaying CNT structure without DA-CMC): (a) 0°, (b) 30°, (c) 60°, (d) 90°, named as DC-0°, DC-30°, DC-60°, and DC-90°.

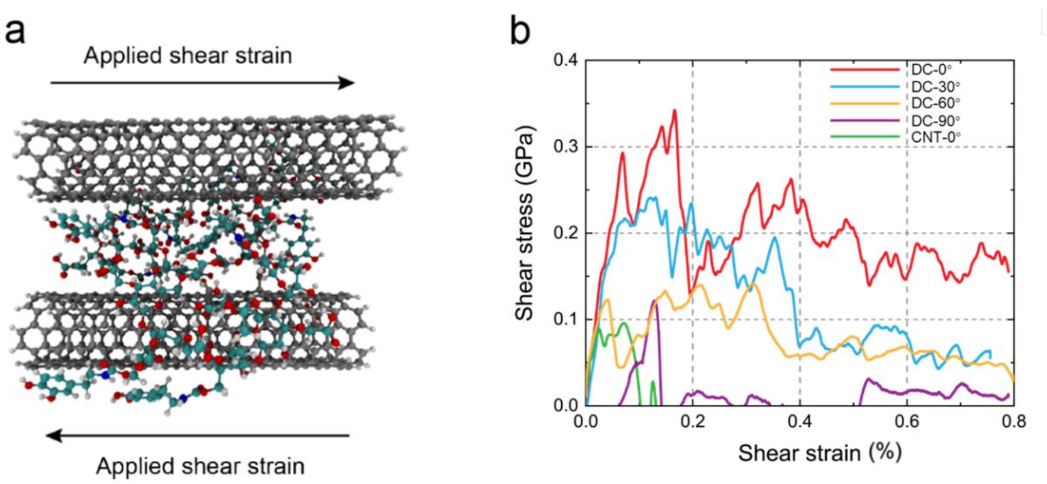

Figure S11. (a) Schematic diagram of DC-0° structure under an applied shear stress. (b) The shear stress-strain curves for composite structures with different skew angles and pure CNT-0°.

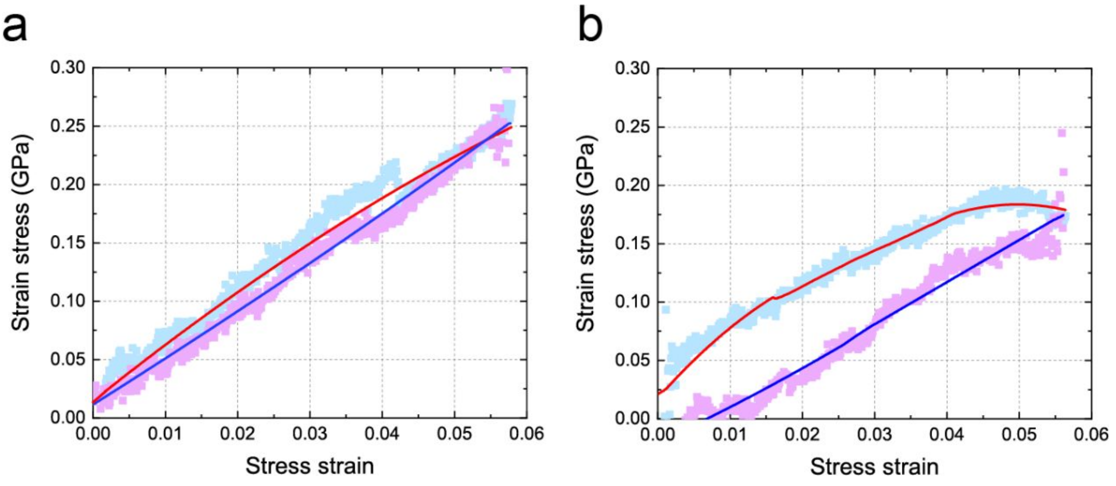

Figure S12. The stress-strain curve of (a) DC-0° and (b) DC-30° undergoing loading-unloading shear stress.

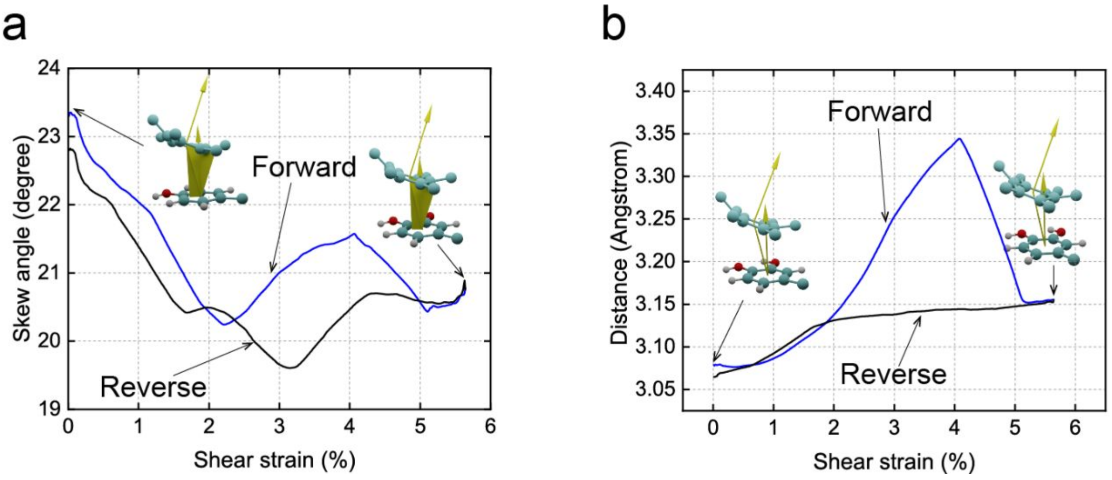

Figure S13. The (a) skew angles variation and (b) distance variation of a catechol in DA-CMC and a carboatomic ring in CNT for DC-30° structure under a cycle of loading-unloading shear stress.

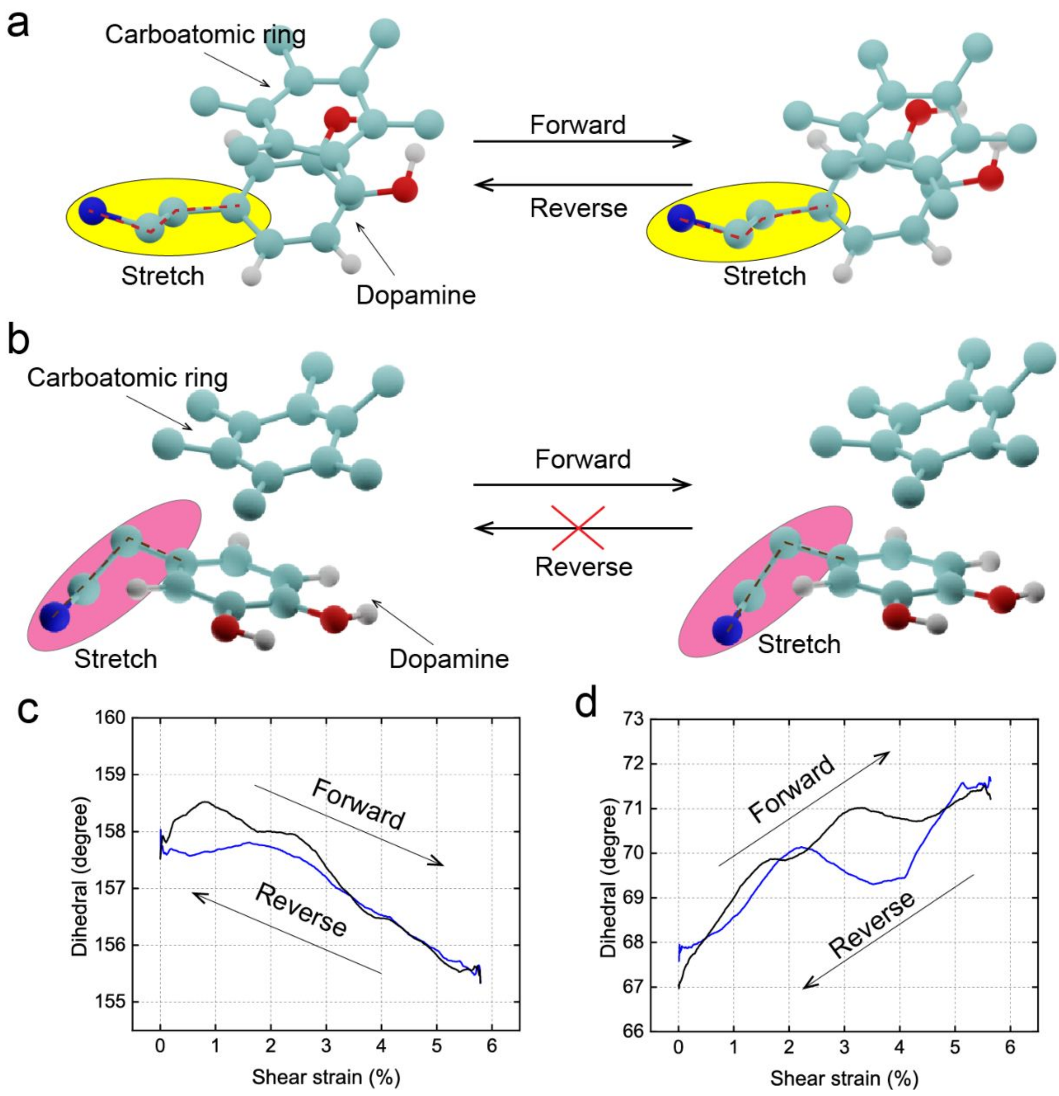

Figure S14. Schematic illustration of dihedral angle (N-C-C-C) evolution of (a) DC-0° and (b) DC-30° under a cycle of loading-unloading shear stress. Dihedral angle change profiles of N-C-C-C for (c) DC-0° and (d) DC-30° under a cycle of deformation.

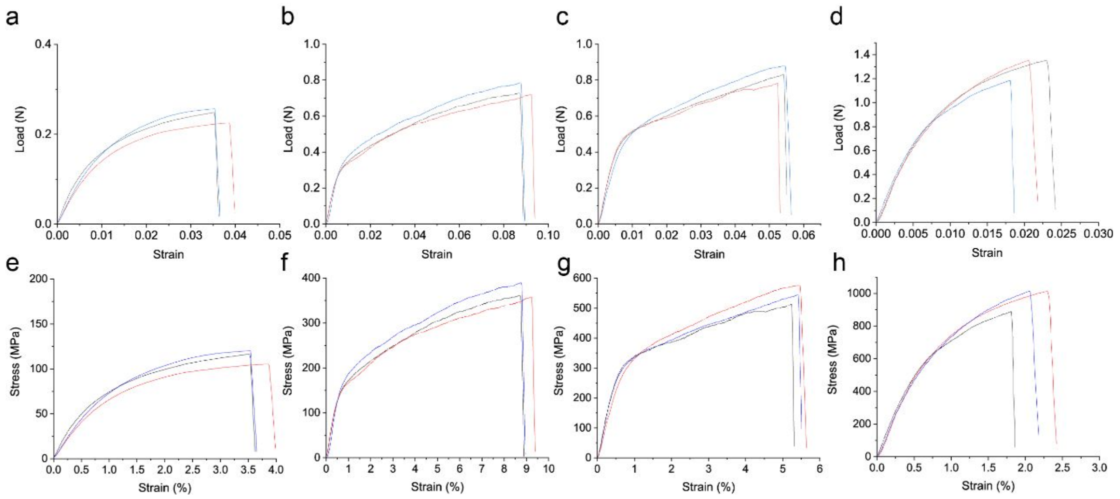

Figure S15. The (a-d) tensile load and (e-h) tensile stress of pure CNT filaments, DA-CMC/CNT@0, DA-CMC/CNT@50, and DA-CMC/CNT@100, respectively.

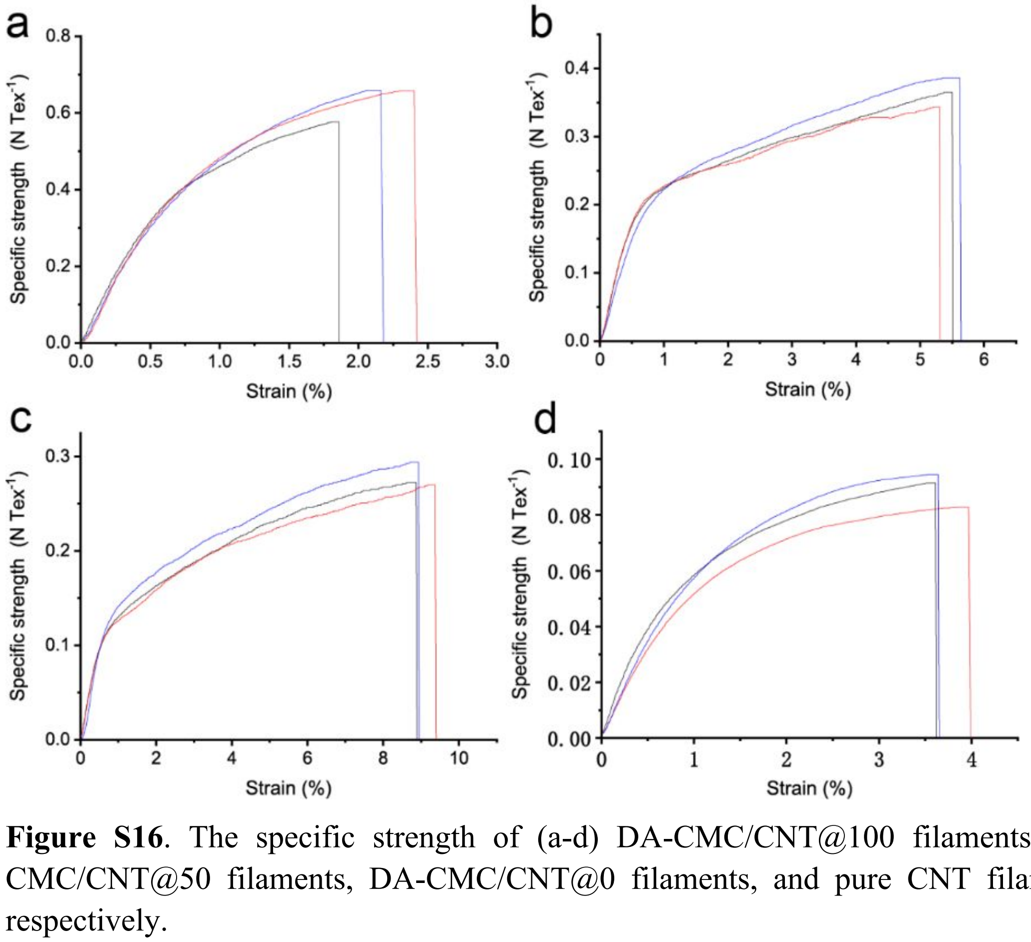

Figure S16. The specific strength of (a-d) DA-CMC/CNT@100 filaments, DA-CMC/CNT@50 filaments, DA-CMC/CNT@0 filaments, and pure CNT filaments, respectively.

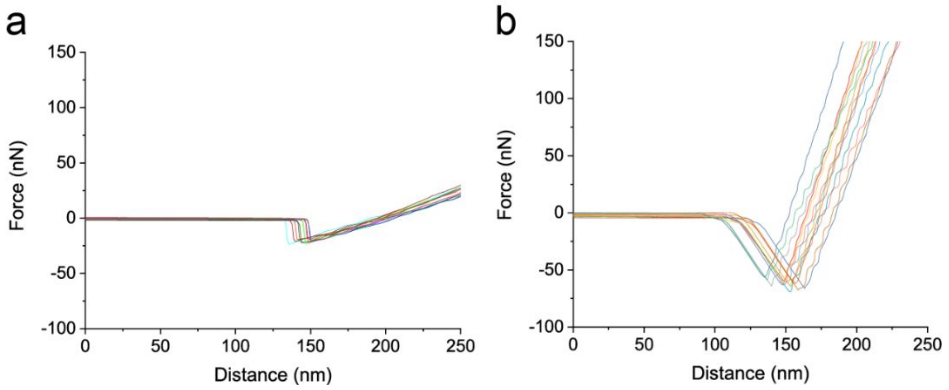

Figure S17. (a) The F-D curves of pure CNT to pure CNT. (b) The F-D curves of DA-CMC/CNT to DA-CMC/CNT.

The total pull-off force of adhesion includes van der Waals interactions, repulsive electrostatic interactions, and additional capillary and chemical bonding,:.[8]

$F_{pull}=F_{vdW}+F_{elec}+F_{cap}+F_{chem}$ (1)

Considering that the samples were composed of a similar structure, without direct chemical bonds between tip and surface, the last two terms can be ignored. Actually, the introduction of DA-CMC can mainly enhance the interfaces through non-bonded interactions, where the interaction between DA-CMC and CNT-COOH is vdW and hydrogen bonding. Besides, the hydrogen bond networks exist within the DA-CMC matrix, which can ensure the strength and toughness of the DA-CMC structure. As is shown in figure a-b, the pull force of the DA-CMC/CNT structure is about 4 times that of pure CNT structure. Also, the elastic modulus of the interfaces, which can be denoted by the slope of the curves, exhibited a similar trend, demonstrating the insertion of DA-CMC does enhance the stiffness of interfaces.

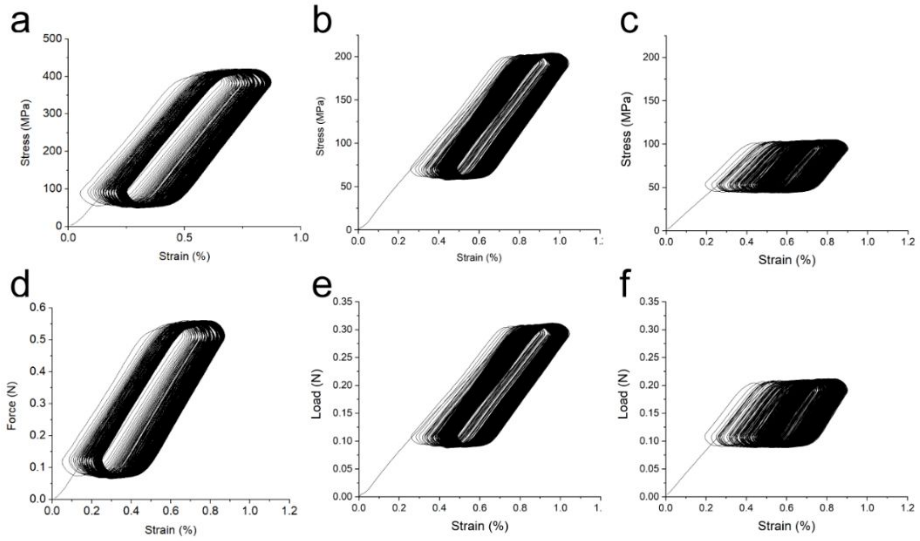

Figure S18. The 1000 loading-unloading cycles of (a-c) stress-strain curves and (d-f) load-strain curves of DA-CMC/CNT@100, DA-CMC/CNT@50, and DA-CMC/CNT@0, respectively.

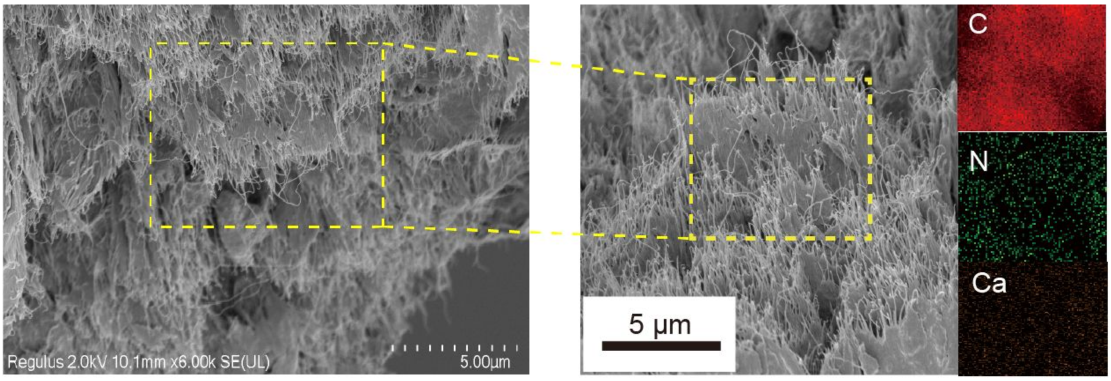

Figure S19. SEM images and EDS maps of DA-CMC/CNT@100 filament's cross section after undergoing 1000 loading-unloading cycles.

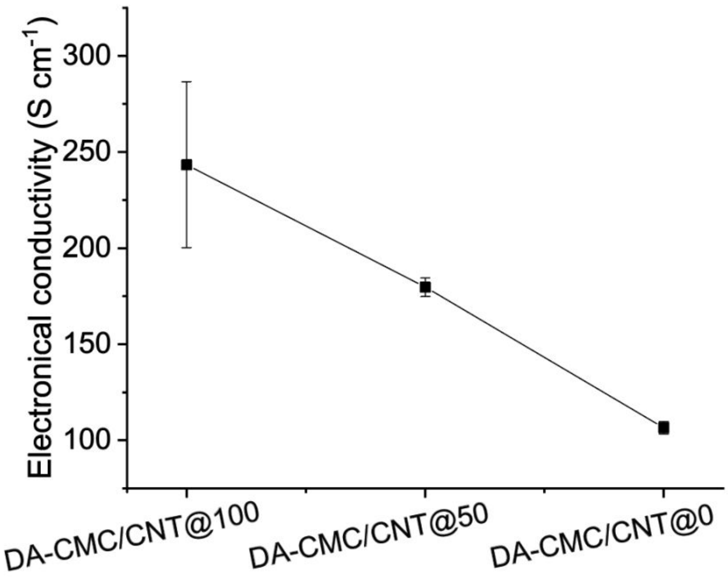

Figure S20. The electrical conductivity of DA-CMC/CNT@100, DA-CMC/CNT@50, and DA-CMC/CNT@0.

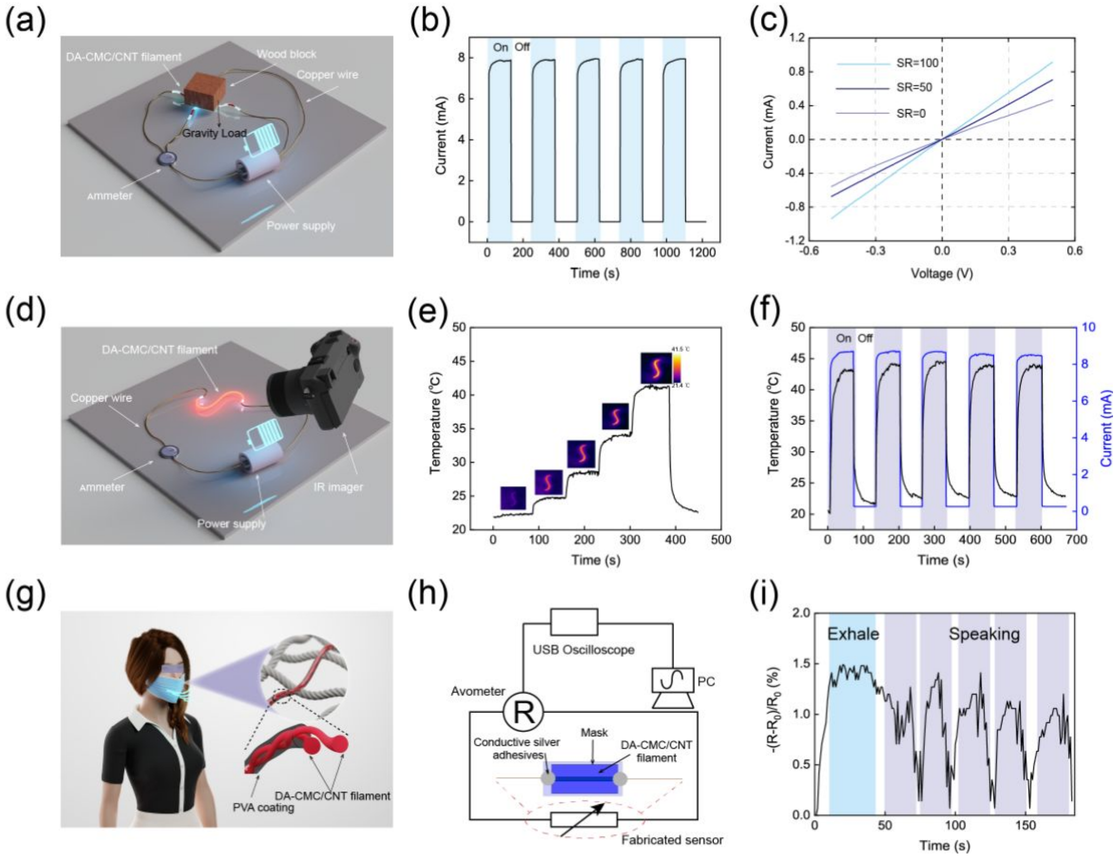

Figure S21. The application of the DA-CMC/CNT@100 filaments. (a) Schematic illustration of composite filaments used as a wire connected to a circuit and supporting a block of wood. (b) Six periodic current cycles at 15 V for composite filaments supporting the woodblock. (c) I-V curve of the composite filament obtained at different SR. (d) Schematic illustration of the electrothermal performance of a composite filament assembled in a “S” shape. (e) The temperature evolution of the composite filaments under an incremental voltage. (f) The temperature and current variation of the composite filament under a cyclic voltage at 15 V. (g) Schematic illustration of a breath sensor and its assembly. (h) Schematic diagram of the breath sensor system based on DA-CMC/CNT filaments. (i) Variation of normalized resistance of composite filaments during use. The periods of exhalation are highlighted with blue color, while the areas corresponding to the process of speaking are highlighted with purple color.

Table S1. The mechanical properties of composite filaments.

Sample	Tensile strength (MPa)	Young's modulus (GPa)	Toughness (MJ m^{-3})	Specific strength (N tex^{-1})	Mass density (g cm^{-3})
DA-CMC/CNT@0	369.26±17.49	25.5±0.13	14.2±2.79	0.63±0.047	1.32±0.031
DA-CMC/CNT@50	543.86±31.42	49.84±3.65	22.35±1.45	0.36±0.021	1.49±0.020
DA-CMC/CNT@100	972.23±72.81	84.14±1.44	24.77±0.92	0.28±0.013	1.54±0.010
Pure CNT	114.04±7.72	8.73±0.92	3.11±0.04	0.089±0.0063	1.27±0.021

Table S2. Data of Ashby plot in Figure 3(f) of the main manuscript

Sample	Ultimate strength (MPa)	Young's Modulus (GPa)	Ref.
CNF/MXene membrane	270	42	9
CNF/SWNT fiber	472	28	10
CNT/PDA fiber	725	83	11
Graphene/PDA fiber	625	125	12
RGO/CNT fiber	690	70	13
Aligned MXene fiber	344	122	14
PVA/CNT fiber	1125	38	15

References used in the construction of the Ashby plot, Figure 4f of the main manuscript:

1. Hess, B.; Kutzner, C.; van der Spoel, D.; Lindahl, E., GROMACS 4: Algorithms for Highly Efficient, Load-Balanced, and Scalable Molecular Simulation. *J. Chem. Theory Comput.* **2008,** *4* (3), 435-447.
2. Wang, J.; Wolf, R. M.; Caldwell, J. W.; Kollman, P. A.; Case, D. A., Development and testing of a general amber force field. *J. Chem. Theory Comput.* **2004,** *25* (9), 1157-1174.
3. Mark, P.; Nilsson, L., Structure and Dynamics of the TIP3P, SPC, and SPC/E Water Models at 298 K. *J. Phys. Chem. A* **2001,** *105* (43), 9954-9960.
4. Hess, B.; Bekker, H.; Berendsen, H. J. C.; Fraaije, J. G. E. M., LINCS: A linear constraint solver for molecular simulations. *J. Chem. Theory Comput.* **1997,** *18* (12), 1463-1472.
5. Humphrey, W.; Dalke, A.; Schulten, K., VMD: Visual molecular dynamics. *J. Mol. Graph.* **1996,** *14* (1), 33-38.
6. Fu, H.; Zhang, H.; Chen, H.; Shao, X.; Chipot, C.; Cai, W., Zooming across the Free-Energy Landscape: Shaving Barriers, and Flooding Valleys. *J. Phys. Chem. Lett.* **2018,** *9* (16), 4738-4745.
7. Fiorin, G.; Klein, M. L.; Hénin, J., Using collective variables to drive molecular dynamics simulations. *Mol. Phys.* **2013,** *111* (22-23), 3345-3362.
8. Leite, F. L.; Bueno, C. C.; Da Róz, A. L.; Ziemath, E. C.; Oliveira, O. N., Theoretical

Models for Surface Forces and Adhesion and Their Measurement Using Atomic Force Microscopy. *Int. J. Mol. Sci.* **2012,** *13* (10).

9. Tian, W.; VahidMohammadi, A.; Reid, M. S.; Wang, Z.; Ouyang, L.; Erlandsson, J.; Pettersson, T.; Wagberg, L.; Beidaghi, M.; Hamedi, M. M., Multifunctional Nanocomposites with High Strength and Capacitance Using 2D MXene and 1D Nanocellulose. *Adv. Mater.* **2019,** *31* (41), e1902977.

10. Wan, Z.; Chen, C.; Meng, T.; Mojtaba, M.; Teng, Y.; Feng, Q.; Li, D., Multifunctional Wet-Spun Filaments through Robust Nanocellulose Networks Wrapping to Single-Walled Carbon Nanotubes. *ACS Appl. Mater. Inter.* **2019,** *11* (45), 42808-42817.

11. Zhang, S.; Hao, A.; Nguyen, N.; Oluwalowo, A.; Liu, Z.; Dessureault, Y.; Park, J. G.; Liang, R., Carbon nanotube/carbon composite fiber with improved strength and electrical conductivity via interface engineering. *Carbon* **2019,** *144*, 628-638.

12. Kim, I. H.; Yun, T.; Kim, J. E.; Yu, H.; Sasikala, S. P.; Lee, K. E.; Koo, S. H.; Hwang, H.; Jung, H. J.; Park, J. Y.; Jeong, H. S.; Kim, S. O., Mussel-Inspired Defect Engineering of Graphene Liquid Crystalline Fibers for Synergistic Enhancement of Mechanical Strength and Electrical Conductivity. *Adv. Mater.* **2018**, e1803267.

13. Eom, W.; Lee, E.; Lee, S. H.; Sung, T. H.; Clancy, A. J.; Lee, W. J.; Han, T. H., Carbon nanotube-reduced graphene oxide fiber with high torsional strength from rheological hierarchy control. *Nat. Commun.* **2021,** *12* (1), 396.

14. Shin, H.; Eom, W.; Lee, K. H.; Jeong, W.; Kang, D. J.; Han, T. H., Highly Electroconductive and Mechanically Strong Ti3C2Tx MXene Fibers Using a Deformable MXene Gel. *ACS Nano* **2021,** *15* (2), 3320-3329.

15. Lee, W. J.; Clancy, A. J.; Fernández-Toribio, J. C.; Anthony, D. B.; White, E. R.; Solano, E.; Leese, H. S.; Vilatela, J. J.; Shaffer, M. S. P., Interfacially-grafted single-walled carbon nanotube / poly (vinyl alcohol) composite fibers. *Carbon* **2019,** *146*, 162-171.
